# Assessing the catastrophic effects of out-of-pocket healthcare payments prior to the uptake of a nationwide health insurance scheme in Ghana

**DOI:** 10.1080/16549716.2017.1289735

**Published:** 2017-05-09

**Authors:** James Akazili, Diane McIntyre, Edmund W. Kanmiki, John Gyapong, Abraham Oduro, Osman Sankoh, John E. Ataguba

**Affiliations:** ^a^ Navrongo Health Research Centre, Ghana Health Service, Navrongo, Ghana; ^b^ Health Systems Working Group, INDEPTH Network, Accra, Ghana; ^c^ Health Economics Unit, School of Public Health and Family Medicine, Faculty of Health Sciences, University of Cape Town, Cape Town, South Africa; ^d^ Office of the Vice Chancellor, University of Health and Allied Sciences, Ho, Ghana; ^e^ School of Public Health, Faculty of Health Sciences, University of the Witswatersrand, Johannesburg, South Africa; ^f^ Department of Mathematics and Statistics, Njala University, Njala, Sierra Leone

**Keywords:** Catastrophic payment, financial risk protection, out-of-pocket healthcare payments, universal health coverage, Ghana

## Abstract

**Background**: Financial risk protection against the cost of unforeseen healthcare has gained global attention in recent years. Although Ghana implemented a nationwide health insurance scheme with a goal of reducing financial barriers to accessing healthcare and addressing impoverishing effects of out-of-pocket (OOP) healthcare payments, there is a paucity of knowledge on the extent of financial catastrophe of such payments in Ghana. Thus, this paper assesses the catastrophic effect of OOP healthcare payments in Ghana.

**Methods**: Ghana Living Standard Survey (GLSS 5) data collected in 2005/2006 are used in this study. Catastrophic effect of OOP healthcare payments is assessed using various thresholds of total household expenditure and non-food expenditure. Furthermore, four indices, namely the catastrophic payment headcount, catastrophic payment gap, weighted catastrophic payment headcount and weighted catastrophic payment gap, are defined and computed.

**Results**: As at 2005/2006, it was estimated that 11.0% of households in Ghana spent over 5% of their total household expenditure on healthcare OOP. However, after adjusting for the concentration of such spending, it decreased to 10.9%. Also 10.7% of households spent more than 10% of their non-food consumption expenditure on OOP healthcare payments. Furthermore, about 2.6% of households are observed to have spent in excess of 20% of their total household income on healthcare OOP. With the exception of the 5% threshold of household expenditure, because the concentration indices of these expenditures are negative, the burden of such expenditures rests more on the poor.

**Conclusions**: Significant levels of financial catastrophe existed in Ghana prior to the uptake of the national health insurance scheme. Poorer households were at a higher risk than the relatively well-off households. The results of this study present baseline assessment of the impact of Ghana’s health insurance policy on catastrophic healthcare payments. Thus, there is a need for continuous monitoring of financial catastrophe in the system to ensure that households are adequately protected.

## Background

The economic consequences of illness in developing countries have gained a lot of global attention in recent years [1–3]. According to the World Health Organization, a concept of fairness in healthcare financing is how households and individuals are protected from, among other things, impoverishing and catastrophic healthcare expenditures [4]. Health shocks such as unpredictable or unforeseeable illnesses that diminish health status are among the most important factors associated with financial catastrophe and impoverishment. Households facing health shocks are often affected by both the payments for medical treatment and income loss due to an inability to work [1]. The situation is even worse for households without any form of health insurance cover as they have a higher risk of incurring substantial expenditures when a member falls ill. Such payments can be catastrophic if they are too large relative to the available household resources to the extent of disrupting the household living standards and limiting their ability to purchase other essential non-medical goods and services [5–8].

Globally, it is estimated that every year about 150 million people incur financial catastrophe while 100 million people are pushed below the poverty line due to out-of-pocket (OOP) healthcare payments [1]. This creates the need for all health systems to ensure access of their populations to universal health coverage (UHC) in such a way that people are shielded from the undue burden of direct OOP payments.

A previous study in Ghana using the 2003 World Health Survey revealed the share of OOP payments in total healthcare expenditure to be 48% [9]. Although this is expected to decrease as the national health insurance expands in coverage, it is significant and has implications on poverty and access to healthcare services. Even in the phase of a nationwide health insurance scheme, OOP payments for healthcare in Ghana are still very prevalent [10].

User fees for public sector health services and direct payments to private sector providers are the two main forms of OOP payments in Ghana. In 1980 user fees were introduced in Ghana through loan conditionalities imposed by the World Bank and the International Monetary Fund (IMF) with their associated Structural Adjustment Programs (SAPs) at that time. Ghana and many other African countries suffered from macroeconomic stagnation as a result of negative growth and increasing indebtedness that resulted from the SAPs [11,12].

The impact of user fees in Ghana was however disastrous as it caused a more than two-thirds drop in utilization of public health services, which was mainly among low-income and vulnerable groups [11,13]. An exemption package that was in place to cushion the effects of user fees on vulnerable populations was poorly implemented due to a lack of clarity and understanding of its operation. The result was that many who were eligible for exemption were not being exempted from paying. A study in the Volta region of Ghana found that as high as 84% of patients who were found to be eligible for exemptions did not receive them [14]. Also, a nationwide study found that nearly half of the patients who were eligible for exemptions were in fact made to pay for services [15].

The consequence of this user fees regime was that people, particularly the poor, were unable to seek needed healthcare services. Whenever such households have to incur healthcare costs, they use coping strategies such as reducing consumption (often on very basic necessities including food), borrowing or selling vital household assets. Indeed people, especially the poor, were ‘detained’ at public health facilities to work in order to pay for the cost of their treatment [15].

Clearly, direct OOP healthcare payments have detrimental impact on the share of households’ disposable income allocated for the consumption of other household basic needs (e.g. food, clothing, utilities, education and shelter) [16]. An equitable and fair health system, therefore, needs to ensure that households do not pay beyond a certain proportion of their total income for health OOP [17,18], especially the poor. Exceeding this proportion would make such payments catastrophic, leading to a reduction in the living standards of such households [17].

While the negative effects of catastrophic payments have been documented globally, there is a paucity of knowledge on the extent and effects of catastrophic OOP expenditures in Ghana. This study attempts to fill this knowledge gap. The primary objective of the paper is to assess the catastrophic effect of healthcare payments in Ghana to highlight the extent to which the health system protects households from the financial consequences of paying OOP for health services. Since the data used in this study were collected in 2005/2006, just around the infancy of national health insurance in Ghana, the results of this study serve as reliable baseline indicators for tracking the trend of catastrophic effects of OOP payments as Ghana’s national health insurance scheme progresses.

## Methods

### Data source

Data for analysis come from the fifth Ghana Living Standard Survey (GLSS 5), which was conducted in 2005/2006. It is a nationally representative survey conducted by the Ghana Statistical Service (GSS) – the national body responsible for conducting all demographic, health and living standard surveys in Ghana. The survey used a two-stage stratified random sampling design. In the first stage a total of 580 enumeration areas were sampled across 10 regions with probability proportional to size. Fifteen households are randomly selected from each enumeration area in the second stage. With an overall response rate in excess of 99%, the final sample size for GLSS 5 was 8687 households, covering a total of 36,488 individuals. These individuals represent 0.17% of the total population of Ghana at the time of this survey. Data collected in the survey relate to all aspects of household decision-making and well-being. The dataset contains information on household consumption of both frequent and non-frequent items (including spending on healthcare). Data on the consumption of non-frequent items were collected for the preceding 12 months whilst those for frequently purchased items were collected weekly for a period of 10 weeks using a weekly diary. In order to ensure consistency, total household expenditure (including OOP spending) used in this paper was annualized. First, frequent household consumption was annualized and added to already annualized non-frequent expenditures. OOP expenditures consist of spending on both inpatient and outpatient services and all other reported spending directly related to the receipt of health services (e.g. medicine, x-rays, laboratory tests, medical equipment). It does not include any part reimbursed by a third party or insurance premiums.

### Data analysis

Stata v11 software was used for all analyses. All estimates were adjusted to reflect national figures using appropriate sampling weights. Total household consumption expenditure was used as a proxy for income. This is a preferred measure because it is less prone to fluctuation and is less likely to be underreported when compared to income [19]. We defined healthcare payment as catastrophic if it exceeds some fraction or threshold of a household’s total expenditure in a given year. We computed results for a range of values for the thresholds (i.e. 5%, 10%, 15%, 20%, 25%, 30%, 35%, 40%) to allow the reader (and policy maker) to make an informed decision rather than limiting it to only thresholds of 10% and 40% for total expenditure and non-food expenditure, respectively. Ten percent and 40% are often cited as representing the points at which the absorption of household resources by spending on healthcare is considered to create severe disruption to living standards [5,20,21]. These are still arbitrary thresholds and they remain a matter of subjective judgment.

## Measuring catastrophic healthcare payments

Let total gross household expenditure be defined as 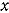, food expenditure as 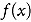 and the catastrophic expenditure threshold be defined as 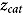, which is a certain proportion of 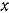. The following indices have been defined for measuring and assessing the catastrophic effect of such healthcare payment [6,17].

### Catastrophic payment headcount index

The catastrophic payment headcount index (Hcat) represents the percentage of households in the population whose healthcare expenditures as a proportion of their income exceed the threshold 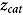. Let 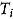 be the OOP healthcare payment of the *^th^*
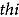
*^th^* household and 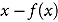 is household non-food expenditure. Catastrophic payment is incurred if the share of household expenditure or non-food expenditure spent OOP exceeds the catastrophic threshold. That is, 
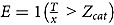
 or 
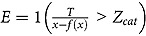
. Empirically the choice of the catastrophic threshold presents a challenge and it often depends on the denominator used for assessing financial catastrophe. In some cases, varying thresholds have been proposed [18].

The incidence of financial catastrophe or catastrophic payment headcount is defined as:

(1)
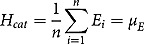

where 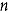 is the total number of households and 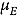 is the mean of 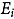.

This measure however does not capture the extent to which the share of household income spent OOP exceeds the threshold. To address this, the catastrophic payment gap is computed.

### Catastrophic payment gap index

This measure captures the average extent to which OOP healthcare payments as a proportion of income exceed the threshold 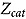. Formally, if we define 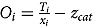 as the extent of overshoot, which is zero for those that do not incur catastrophic payments,  then the catastrophic payment gap may be specified as

(2)
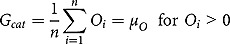

where 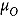 is the mean of 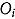.

Because the catastrophic payment gap is averaged across all households, irrespective of their financially catastrophic status, it does not adequately reflect the extent to which such payments are substantial among those incurring them. So, the mean positive gap (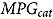) defined in Equation (3) is computed to reflect this. This basically reflects the average catastrophic gap among those that incur financial catastrophe [17,18] (i.e. households used to compute the headcount in Equation (1)):

(3)
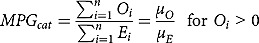

The approaches outlined previously are blind as to whether it is the poor or the better-off households that exceed the threshold [6,17]. It can be argued that most societies including Ghana will worry more if it is households in the lowest quintile that incur financial catastrophe more than richer households. While there may be other ways of addressing this [18], this paper constructs a weighted version of all the indices (headcount and gap) that takes into consideration whether it is the better-off or mostly the poor households that exceed the threshold. While this represents one approach, an alternative is to deviate from using a fixed threshold to have varying thresholds where poorer households, relative to richer households, have lower threshold values [18].

### Weighted catastrophic payment headcount and weighted catastrophic gap indices

In order to obtain these indices, the concentration indices  of 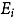 and 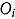 are computed such that 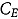 is the concentration index of 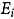 and 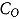 is the concentration index of 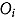. A negative concentration index (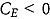 or 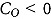) means that such payments are concentrated among poorer households.

Weighted catastrophic payment headcount is obtained as:

(4)
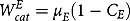

If 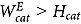 it means that poorer households exceed the threshold more but if 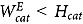 then the incidence of catastrophic spending is concentrated on the rich [5,17]. Basically, if poorer households tend to exceed the threshold more, the concentration index 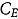 will be negative and this will raise 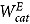 above 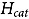. The reverse is the case when 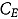 is positive [17].

Weighted catastrophic payment gap on the other hand is obtained as:

(5)
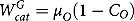

If 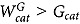 then large excesses tend to be concentrated among the poor but the reverse is the case when 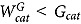 [6].

Table 1 contains a summary of the different techniques for assessing financial catastrophe.

**Table 1.** Summary of the different techniques of measuring catastrophic payments and their limitations.

Types of measures		Technique(s) of measurements	Limitation(s)
1	Catastrophic payment headcount index,	Total household OOP healthcare expenditures as a proportion of their income exceeding a given threshold	This does not capture the height above which households exceed the threshold
2	Catastrophic payment gap index,	Measure captures the average degree by which total household OOP healthcare payments as a proportion of household income exceed the threshold	Blind to whether it is the poor or the better-off households that exceed the threshold
3	Weighted catastrophic payment headcount index,	Weighted version of the headcount that takes into consideration whether it is the better-off or mostly poorer households that exceed the threshold	Although the original headcount changes, it is not useful for identification [18]
4	Weighted catastrophic payment gap index,	Measures the tendency of large excesses (overshoot) of catastrophic payments to be concentrated among the worst-off or better-off	As above. Also blind as to how far catastrophic payment causes hardship to individuals or households

Ethical clearance for this study was obtained from the Ghana Health Services ethics review board and the University of Cape Town Human Research Ethics Committee.

## Results

In developing countries like Ghana, payment for healthcare can crowd out food expenditure relative to total expenditure. Therefore results for both total expenditure and non-food expenditure are presented. The percentage of households incurring catastrophic payment necessarily falls as the threshold is raised, irrespective of the index used in estimation. This shows that the incidence and intensity of catastrophic healthcare payments are a function of the threshold used.

Table 2 shows the catastrophic healthcare payments computed for various thresholds for the stated indices using total household expenditure and household non-food expenditure. As shown in the table, 11.0% of Ghanaian households spent in excess of 5% of total household expenditures on healthcare in 2005/2006. At a threshold of 10%, the number of households reduces by half. About 2.6% of households are observed to have spent in excess of 20% of the total household expenditures.

**Table 2.** Catastrophic healthcare payment in Ghana, 2005/2006.

	Catastrophic healthcare payment using total household expenditure				Catastrophic healthcare payment using household non-food expenditure			
Thresholds	5%	10%	15%	20%	10%	20%	30%	40%
Headcount								
Catastrophic payment headcount ()	11.00%	5.16%	3.39%	2.56%	10.70%	4.91%	3.17%	2.43%
Concentration index []	0.012	–0.016	–0.050	–0.065	–0.019	–0.045	–0.080	–0.087
Weighted headcount ()	10.87%	5.24%	3.56%	2.72%	10.90%	5.13%	3.42%	2.64%
Gap measures								
Catastrophic payment gap ()	1.83%	1.47%	1.26%	1.11%	3.39%	2.68%	2.29%	2.01%
Concentration index []	–0.048	–0.061	–0.066	–0.068	–0.104	–0.122	–0.132	–0.139
Weighted gap ()	1.92%	1.56%	1.34%	1.19%	3.75%	3.01%	2.59%	2.29%
Mean positive gap ()	16.66%	28.47%	37.22%	43.66%	31.72%	54.57%	72.26%	82.62%

Notes: All indicators are weighted to reflect national figures. A total of 8604 households are used in this analysis.

Furthermore, it can be observed that at the 5% proportion of total income threshold, when the concentration of catastrophic payments is taken into account, about 10.9% of the population incurs catastrophic total healthcare expenditure. The values decline to 2.7% at the 20% threshold. It is important to note that the weighted headcount (10.9%) at the 5% threshold of total expenditure is slightly lower than the original headcount (11.0%). This means that richer households are making slightly more catastrophic payments at this threshold (which is confirmed by the positive concentration index). However, at higher thresholds of total household expenditure, the poor are more often faced with catastrophic health expenditure, as reflected by the negative concentration indices. Also for the catastrophic headcount using non-food expenditure, 10.7% of households spent more than 10% of their non-food consumption expenditure OOP on healthcare. Results from using total non-food expenditure as the denominator show that the poor are burdened more with catastrophic expenditure as reflected in the negative concentration indices (–0.019, –0.045, –0.080 and –0.087 at the 10%, 20%, 30% and 40% thresholds of household non-food expenditure, respectively).

## Discussion

The financially catastrophic effects of healthcare payments have been examined using a cross-sectional nationwide dataset collected in 2005/2006 at a time when Ghana’s national health insurance scheme had just been introduced. The results show that a high percentage of Ghanaian households incurred financial catastrophe irrespective of the threshold considered.

With 11.0% of households spending in excess of 5% of their total expenditure on healthcare, this is higher when compared to other developing countries (e.g. Malaysia, Philippines and Thailand). In 1998/99 for instance, only 7% of Malaysian households spent in excess of 5% of their total household income on healthcare OOP. In the same year, using the poverty indicator survey, it revealed that in the Philippines less than 10% of households spent in excess of 5% of their total household income on healthcare [17,22]. Less than 9% of Thailand’s households spent in excess of 5% of their total household income on healthcare in 2002 [22]. However, higher figures were observed in China and Vietnam. For instance, 28% of Chinese households recorded OOP payments in excess of 5% of their total income while Vietnam recorded 38% of households in 1993 that had incurred catastrophic payment at the 5% threshold level [17,22]. A study in Nigeria revealed that as many as 39% of households recorded OOP healthcare payments in excess of 5% of their total household income [23].

The results from this study also show a positive concentration index for the catastrophic headcount at the 5% threshold of total household income. However, the concentration indices were negative for higher thresholds. Thus, the weighted headcount at the 5% threshold was lower than that of the unweighted. This result means that the share of total income that poorer households spend OOP on health services tended to be very high in Ghana, with catastrophic payments concentrated more among the poor at higher thresholds of total household income. This also raises the issue of the choice of an appropriate threshold for assessing financial catastrophe. While this paper is unable to provide an answer as to the most appropriate threshold, the results clearly indicate that the choice of thresholds matters as seen in the case of Ghana.

The percentage of households in a country spending in excess of 5% of total household expenditure on healthcare seems to be indirectly influenced by general economic development. Ghana’s figure of 11.0% households incurring OOP payments in excess of 5% of expenditure reflects the modest economic growth that resulted in overall poverty reduction in the country over the previous decade. The economy recovered from negative GDP growth in the early 1980s to over 6% GDP growth in 2007/2008 [24,25].

It is evident from the results presented in this paper that non-food expenditure absorbs a considerable share of household resources given that 10.7% of households spent more than 10% of their non-food consumption expenditure on healthcare. In Malaysia only 1% of households spent in excess of 10% of their non-food consumption on healthcare [22]. Once basic food needs have been met, healthcare costs can account for a large portion of resources for a substantial fraction of the population. In similar studies in Asia, between 8% and 16% of households in countries like Nepal, Vietnam, Kyrgyzstan and Bangladesh spend in excess of 25% of non-food expenditure on OOP payments [6,22]. The degree of poverty in Nepal and Kyrgyzstan implies that food absorbs a very large share of household resources and reduces the share of total resources that can be devoted to healthcare.

For catastrophic healthcare payments using household non-food expenditure, this paper shows that the poor are burdened more with catastrophic expenditure. This is confirmed by the negative concentration indices, which make the values of the weighted headcount higher than those of the unweighted headcount. These results are to be expected given that food expenditures take a larger share of the resources in poorer households. Similar results are reported in China, South Korea, Nepal, Sri Lanka, Vietnam and Taiwan, where poorer households were more likely to incur high catastrophic expenditure than richer households [22].

These findings in Ghana, where poorer households were more likely than higher-income groups to make catastrophic healthcare payments at higher thresholds, reflected the weak implementation of the poverty reduction strategies and more importantly the user fee exemption package. The exemption policy in the health sector was introduced to cushion the effects of user fees in the 1980s. It was meant to exempt the poor, the aged (70+), children under five and antenatal care recipients from paying OOP. But inefficiency and lack of ability to identify the poor, the aged etc. hindered the effective implementation of this exemption program [26]. Where exemptions were more effectively implemented in countries like Malaysia, the Philippines, Indonesia and Thailand, catastrophic payments are made disproportionately by the better-off groups [22].

It is important to note that both the prevalence and the intensity are reflected in the catastrophic payment gap. From the results of the catastrophic gap, the mean positive gap, the concentration indices and the weighted gap, it can be observed that the indices of the gap and the weighted gap decline in both the total household expenditure and non-food expenditure measures as we move from a lower to a higher threshold. The negative values of the concentration index show that it is the poor who are more likely to incur healthcare payments exceeding the thresholds. The intensity of catastrophic payments thus affects the poor more than the rich for both the total household expenditure and non-food expenditure measures. The results of the mean overshoot are substantial, suggesting that there is a high intensity of catastrophic health expenditure among households incurring such expenditures, especially among the poor, and it is more pronounced with non-food expenditure. For instance, among those devoting more than 20% of total expenditure to OOP payments on healthcare, the average OOP payment share exceeds this threshold by almost 44 percentage points, giving a significant OOP budget share of 64%. The average budget share for those exceeding the 20% of non-food expenditure threshold is much higher – about 75% OOP budget shares. This reflects a higher percentage of food expenditure in total household expenditure especially among the poor. From Nepal’s 1999/2000 household expenditure survey, among those spending more than 25% of their non-food expenditure on OOP payments, the average OOP budget share exceeded this by 34 percentage points, giving a 59% OOP budget share. It was a 44% OOP budget share in Bangladesh [22]. The findings point to a high intensity of catastrophic payment in Ghana, exceeding that observed in countries like Nepal and Bangladesh [22]. It is relevant for policy makers to know whether it is the rich or the poor that are more likely to overshoot these thresholds. As shown in this paper, poorer households in Ghana are more likely to overshoot the thresholds with respect to both total household expenditure and non-food expenditure on all the thresholds. This is highlighted by the negative concentration index of the catastrophic gap and the relatively higher weighted catastrophic payment gap (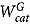). In contrast to these findings, the rich or better-off are more likely to overshoot than poorer households in many of the Asian countries [22]. A study in Nigeria also revealed a tendency for catastrophic healthcare expenditure to be more prevalent among richer households [23]. The findings of the intensity of catastrophic payments indicate the regressive nature of OOP payment.

A major strength of the paper is the use of nationally representative data for assessing financial catastrophe in Ghana. Also, the data on OOP spending are comprehensive because the weekly diary method was used for data collection. However, the study has some limitations. A major limitation to survey-based measures of catastrophic financial risk assessments is the tendency to understate the risk faced by poorer households that are unable to seek care because of cost (and thus reported zero health expenditures). Another limitation is the fact that illness shocks have catastrophic economic consequences not only through medical expenses but also as a result of lost earnings. These limitations notwithstanding, healthcare spending in excess of a substantial fraction of household resources is informative, at least with respect to part of the catastrophic economic consequences of illness even if it does not fully address the welfare losses from a lack of financial risk protection against health shocks [22]. Because the data used in this study come from a previous GLSS round, there is need for future studies that use recent datasets, as soon as they are available, to continue to monitor the pattern of catastrophic health expenditures and indeed financial risk protection in Ghana as the country progresses towards UHC.

The results of this paper also speak to the debilitating nature of OOP healthcare payments and provide strong reasons why Ghana and other developing countriesthat rely heavily on OOP payments, which are often very regressive, should quickly move to a pre-payment system in the form of a comprehensive health insurance. This will not only provide sound financial risk protection to the population but will position countries to move towards universal healthcare coverage. This information will surely push policy makers and planners to redouble their efforts to ensure that everybody, especially the poor and vulnerable populations, is adequately covered by a pre-payment system like the national health insurance scheme in Ghana. Even within the national health insurance regime it will still be important to continue to analyse national survey data to see if the national health insurance scheme is helping to reduce the incidence of catastrophic and impoverishment effects of healthcare payments.

## Conclusion

Overall, it is to be noted that OOP payment has a catastrophic effect on Ghanaians and poorer households are at a higher risk of financial catastrophe. Comparing Ghana with selected African and Asian countries, Ghana was worse off in terms of having a higher catastrophic effect of OOP payments (using both the 10% of total expenditure and the 40% of non-food expenditure thresholds) compared to Tanzania, South Africa, Malaysia, Sri Lanka, Thailand and Indonesia. However, Ghana is better off than Nepal, China and Bangladesh. Countries that have higher catastrophic healthcare payments are countries which are also associated with significant OOP payments. The results of this study present baseline indicators for assessing the impact of Ghana’s health insurance policy on catastrophic healthcare payments.

## Paper context

The paper assesses the catastrophic effect of out-of-pocket health care payments in Ghana prior to the significant roll out of the national health insurance scheme. Significant levels of financial catastrophe existed in Ghana. Poorer households were at the higher risk than relatively well-off households. The paper presents a baseline assessment of the impact of Ghana National health insurance policy on catastrophic health care payments.
